# Risk analysis for the reintroduction and transmission of measles in the post-elimination period in the Americas

**DOI:** 10.26633/RPSP.2017.157

**Published:** 2017-12-20

**Authors:** Daniele Rocha Queiroz Lemos, Aidee Ramirez Franco, Marcio Henrique de Oliveira Garcia, Desiree Pastor, Pamela Bravo-Alcantara, Jose Cassio de Moraes, Carla Domingues, Luciano Pamplona de Goes Cavalcanti

**Affiliations:** 1 Faculty of Medicin Centro Universitário Christus Fortaleza, CE Brazil Faculty of Medicine, Centro Universitário Christus, Fortaleza, CE, Brazil.; 2 Pan American Health Organization Pan American Health Organization Washington, D.C. United States of America Pan American Health Organization, Washington, D.C., United States of America.; 3 Ministry of Health Ministry of Health Brasília, DF Brazil Ministry of Health, Brasília, DF, Brazil.; 4 Faculty of Medical Sciences Santa Casa de Misericórdia São Paulo, SP Brazil Faculty of Medical Sciences, Santa Casa de Misericórdia, São Paulo, SP, Brazil.; 5 Faculty of Medicine Universidade Federal do Fortaleza Fortaleza, CE Brazil Faculty of Medicine, Universidade Federal do Fortaleza, Fortaleza, CE, Brazil.

**Keywords:** Epidemiology, risk assessment, disease eradication, Brazil., Epidemiología, medición de riesgo, erradicación de la enfermedad, Brasil, Epidemiologia, medição de risco, erradicação de doenças, Brasil

## Abstract

**Objective.:**

*To propose and test a model for analyzing municipalities’ level of risk of reintroduction and transmission of the measles virus in the post-elimination period in the Americas*.

**Methods.:**

*An ecological-analytical study was conducted using data on the measles epidemic that occurred in 2013–2015 in northeastern Brazil. The variables for analysis were selected after an extensive review of scientific literature on the risk of importation of measles cases. A univariate analysis considering the presence or absence of confirmed cases of measles in 184 municipalities in the state of Ceará, Brazil, was carried out to evaluate the association between the dependent variable and 23 independent variables, grouped into four categories: 1) characteristics of the municipalities; 2) quality indicators for immunization programs and epidemiological surveillance; 3) organizational structure for the public health response; and 4) selected impact indicators. A* P *value < 0.05 was considered significant. All variables with* P *< 0.200 were analyzed using multivariate logistic regression. Based on the results, the municipalities were categorized by four levels of risk (“low,” “medium,” “high,” and “very high”)*.

**Results.:**

*The model sensitivity was 95% for concordance between municipalities classified as “high risk” and “very high risk” and those that had an epidemic between 2013 and 2015 in Ceará. Of the 38 municipalities that had an epidemic, 76% (29/38) were classified as “high risk” and “very high risk”; 146 municipalities did not report cases (*P *< 0.0002)*.

**Conclusions.:**

*Given the imminent risk of reintroduction of measles circulation in the post-elimination period in the Americas, this model may be useful in identifying areas at greater risk for reintroduction and continued transmission of measles. Knowledge of vulnerable areas could trigger appropriate surveillance and monitoring to prevent sustained transmission*.

## RESUMEN

**Objetivo.:**

Proponer y poner a prueba un modelo para analizar el nivel de riesgo de reintroducción y transmisión del virus del sarampión que existe en los municipios durante el período posterior a la eliminación en la Región de las Américas.

**Métodos.:**

Se realizó un estudio ecológico y analítico empleando datos sobre la epidemia de sarampión que afectó al noreste del Brasil del 2013 al 2015. Las variables para el análisis se seleccionaron después de efectuar un amplio examen de las publicaciones científicas sobre el riesgo de importación de casos de sarampión. Se llevó a cabo un análisis con una sola variable considerando la presencia o ausencia de los casos de sarampión confirmados en 184 municipios del estado de Ceará (Brasil) para evaluar la asociación entre la variable dependiente y 23 variables independientes, que se agruparon en cuatro categorías: 1) características de los municipios; 2) indicadores de calidad de los programas de vacunación y la vigilancia epidemiológica; 3) estructura de organización de la respuesta de salud pública, y 4) indicadores del impacto seleccionados. Se consideró significativo un valor de *P* < 0,05. Todas las variables con un valor *P* < 0,200 se analizaron empleando una regresión logística con varias variables. Teniendo en cuenta los resultados, los municipios se clasificaron en función de cuatro niveles de riesgo (“bajo”, “medio”, “alto” y “muy alto”).

**Resultados.:**

El modelo tenía una sensibilidad de 95% en el caso de la concordancia entre los municipios clasificados dentro de las categorías de “riesgo alto” y “riesgo muy alto” y los que tuvieron una epidemia entre el 2013 y el 2015 en Ceará. De los 38 municipios que tuvieron una epidemia, 76% (29/38) se clasificaron dentro de las categorías de “riesgo alto” y “riesgo muy alto”; 146 municipios no notificaron casos (*P* < 0,0002).

**Conclusiones.:**

Dado el riesgo inminente de reintroducción de la circulación del sarampión durante el período posterior a la eliminación en la Región de las Américas, este modelo puede ser útil para reconocer las zonas en las que existe un mayor riesgo de reintroducción y transmisión continua del sarampión. El conocimiento de las zonas vulnerables podría desembocar en actividades de vigilancia y seguimiento apropiadas para evitar la transmisión sostenida.

## RESUMO

**Objetivo.:**

Elaborar e testar um modelo para analisar o risco de reintrodução e transmissão do vírus do sarampo ao nível municipal no período pós-eliminação nas Américas.

**Métodos.:**

Um estudo analítico-ecológico foi realizado com base nos dados da epidemia de sarampo ocorrida em 2013–2015 no nordeste do Brasil. As variáveis para análise foram selecionadas após extensa revisão da literatura científica sobre o risco de importação de casos de sarampo. Uma análise univariada considerando a presença ou a ausência de casos confirmados de sarampo em 184 municípios no Estado do Ceará foi conduzida para avaliar a associação entre a variável dependente e 23 variáveis independentes divididas em quatro grupos: 1) características dos municípios, 2) indicadores de qualidade dos programas de vacinação e da vigilância epidemiológica, 3) estrutura organizacional para resposta em saúde pública e 4) indicadores de impacto selecionados. Um nível de significância de 5% foi definido. Todas as variáveis com *P* < 0,200 foram analisadas por regressão logística multivariada e, segundo os resultados, os municípios foram categorizados em quatro níveis de risco: baixo, intermediário, alto e muito alto.

**Resultados.:**

A sensibilidade do modelo foi de 95% para concordância entre os municípios categorizados como “risco alto” e “risco muito alto” e os que registraram a ocorrência de epidemia entre 2013 e 2015 no Ceará. Dos 38 municípios onde ocorreu uma epidemia, 76% (29/38) apresentaram “risco alto” e “risco muito alto” de reintrodução e transmissão do vírus do sarampo e 146 municípios não notificaram casos (*P* < 0,0002).

**Conclusão.:**

Diante do risco iminente de reintrodução da circulação do vírus do sarampo no período pós-eliminação nas Américas, este modelo pode servir para identificar as áreas de maior risco de reintrodução e transmissão contínua do vírus do sarampo. Conhecer as áreas vulneráveis incentiva a adoção de procedimentos adequados de vigilância e monitoramento a fim de prevenir a transmissão sustentada.

Measles continues to be one of the main causes of morbidity and mortality among children under 5 years old, especially in malnourished children and those living in less economically developed countries (1). In 2015, about 250 000 cases of measles, resulting in 184 000 deaths (about 400 per day), were registered worldwide (2, 3). Indigenous transmission of measles virus in the Americas was interrupted in 2002 and declared eliminated by the Pan American Health Organization (PAHO)/World Health Organization (WHO) in 2016 (4–6). After interruption of indigenous transmission, imported cases were confirmed in Brazil, Canada, Ecuador, and the United States (6). In 2013, the measles virus was reintroduced in Brazil, with about 1 200 confirmed cases in Permanbuco and Ceará, two northeastern states (7, 8).

Given the need for sustainable elimination of the disease in the Americas, and taking into account the new context and challenges of the post-elimination period, two strategic plans were developed and implemented and remain in force until 2020—a global plan, and a Regional plan (9–11).

A recent study has developed a tool to measure the risk of dissemination of measles in regions close to elimination^a^ (12). The objective of this study was to propose and test the model for analyzing municipalities’ level of risk of reintroduction and transmission of the measles virus in the post-elimination period in the Americas. The model analyzed data on the measles epidemic in northeastern Brazil between 2013 and 2015.

## MATERIAL AND METHODS

### Description of study and the selection and analysis of variables

This study was ecological and analytical (13). The variables were selected after an extensive review of scientific literature on the risk of importation of measles cases. The municipality was the unit of analysis, according to the International Classification of Diseases, 10^th^ revision (ICD-10) and PAHO/WHO recommendations. The scenario of the year before the occurrence of a measles epidemic in Brazil’s northeastern state of Ceará was analyzed. The occurrence or nonoccurrence of measles cases in 184 municipalities in that state was the dependent (outcome) variable. The selected independent variables (a total of 23) were grouped into four categories: 1) municipality characteristics; 2) quality indicators for immunization programs and epidemiologic surveillance; 3) organizational structure for the public health response; and 4) impact indicators (Table 1). The independent variables were dichotomized (“presence” versus “absence”), using medians and values defined as adequate by the Brazilian Ministry of Health and Ministry of Tourism, and PAHO/WHO, as cutoff points.

**TABLE 1.** Odds ratios for four categories of health-related variables (*n* = 23) assessed as municipality risk factors for reintroduction/transmission of measles in a post-elimination scenario, based on their association with the presence or absence of confirmed measles cases in 184 municipalities during an epidemic in Ceará, Brazil, 2013–2015

Variables^a^	P	OR^b^	CI^c^
Municipality characteristics			
1. Tourism^d^	0.0008	3.49	1.70–9.13
2. Population density index^d^	0.0011	0.30	0.14–0.63
3. Urbanization^d^	0.0024	3.93	1.55–9.99
4. Municipality own resources spent on health	0.3182	0.68	0.32–1.43
5. Health expenditures per inhabitant	0.4675	0.76	0.37–1.56
6. Industrialization	0.2441	0.65	0.31–1.34
7. Violence	0.5292	0.77	0.34–1.71
8. Municipal Human Development Index (MHDI)	0.3950	0.15	0.98–1.13
9. Presence of vulnerability conditions^e^	–	–	–
Quality indicators for immunization programs and epidemiologic surveillance			
10. MMRf dose ^f^coverage at 12 months 0.3729	0.3729	1.38	0.67–2.83
11. Dropout rate between MMR dose 1 and 2^d^	0.0066	3.71	1.85–16.4
12. Notification of exanthematic diseases	0.2527	1.58	1.71–3.46
13. Vaccines of regular scheme up-to-date in children < 1 year old	0.2803	1.84	0.60–5.64
14. Vaccines of regular scheme up-to-date in children 1–2 years old	0.3102	0.68	0.33–1.41
15. Coverage of follow-up campaign in 2011 (children 1–6 years old)	0.4924	1.42	0.51–3.89
16. Dropout rate between dose 1 of PENTA^g^and dose 1 of MMR	0.7352	0.87	0.40–1.89
17. Homogeneity	0.4795	0.76	0.37–1.59
Organizational structure for the public health response			
18. Community Health Agent coverage^d^	0.0020	7.22	1.64–31.7
19. Family Health Strategy (FHS) coverage^d^	0.0001	3.52	1.62–7.63
Impact indicators			
20. Malnutrition in children < 1 year old	0.2540	1.51	0.74–3.10
21. Malnutrition in children 1–2 years old	0.3102	0.68	0.33–1.41
22. Children < 4 months old with exclusive breastfeeding	0.6695	0.85	0.41–1.75
23. Child mortality rate	0.4150	0.74	0.36–1.51

***Source:*** Prepared by the authors based on the study results.

a Information about the variables used in the model are accessible online from the following sources: 1) the information system of Brazil’s publicly funded health care system, DATASUS (*Departamento de Inform ática do Sistema Único de Saúde*), the entity responsible for collecting, processing, and disseminating health information; 2) the National Immunization Program Information System (*Sistema de Informação do Programa Nacional de Imunização*, SI-PNI); 3) the Notification of Injury Information System (*Sistema de Informação de Agravos de Notificação*, SINAN); 4) the Brazilian Institute of Geography and Statistics (*Instituto Brasileiro de Geografia e Estatística*, IBGE); 5) Cear á Research Institute on Economic Strategy (*Instituto de Pesquisa e Estratégia Econômica do Cear á*, IPECE); 6) the Latin American School of Social Sciences (*Facultad Latinoamericana de Ciencias Sociales*, FLACSO) (the “Violence Map”); 7) the Brazilian Ministry of Tourism (the“Tourism Map”); and 8) the Department of Primary Care of the Brazilian Ministry of Health (*Departamento de Atenção B ásica, Secretaria de Atenção à Saúde*, DAB-MS).

b OR: odds ratio.

c CI: confidence interval.

d *P* value < 0.05.

e Border with other countries, favelas, violence, indigenous communities, population resistant to vaccination, difficult geographic access; and areas with trade fairs and mass events. This variable was originally included in the analysis to comply with the criteria for PAHO’s recommendation on regions that should be intensively monitored for the introduction of the measles virus, and was part of the second model extract the second extract.

f Measles-mumps-rubella vaccine.

g Pentavalent vaccine (protection against five diseases: diphtheria, pertussis, tetanus, hepatitis B, and Haemophilus influenzae type B (Hib)).

A univariate analysis was carried out to evaluate the association between the 23 independent variables and the dependent variable (occurrence or nonoccurrence of measles. In this first step of the analysis, variables with a *P* value < 0.05 based on the chi-square test or Fisher’s exact test—a total of six— were considered statistically significant indicators of risk and were incorporated into the model for multivariate analysis. All variables with a *P* value < 0.200 were analyzed using multivariate logistic regression. In the second step of the analysis, three additional variables were added: 1) coverage with dose 1 of the measles-mumps-rubella vaccine (MMR); 2) the notification rate for febrile eruptive (exanthematic) diseases; and 3) the presence of vulnerabilities (border with other countries, *favelas* (shanty towns), violence, indigenous communities, population resistant to vaccination, difficult geographic access, and areas with trade fairs and mass events).

**FIGURE 1.**
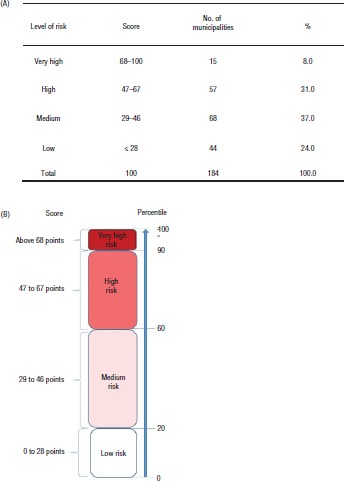
Level of risk of measles reintroduction/transmission post-elimination based on total score for selected indicators (A), and total score by percentile (B), using data from a post-elimination scenario for 184 municipalities with and without confirmed measles cases during an epidemic in Ceará, Brazil, 2013–2015

***Source:*** Prepared by the authors based on the study results.

Each municipality received a total score of 0 to 100. After weighting the variables and scores, the municipalities were classified as “low risk,” “medium risk,” “high risk,” or “very high risk,” using the 20th, 60th, and 90th percentiles to establish cutoff points. Thirteen points were given for each variable in the first extract (for the variables with statistical significance) except “coverage by Community Health Agents (CHAs),” which had a higher odds ratio (7.22) so was worth 14 points. Municipalities with a total score of 28 points or less were classified as “low risk,” whereas those with 29-46 points were classified as “medium risk,” those with 47-67 points were classified as “high risk,” and those with scores of 68 or higher were classified as “very high risk” (Figure 1).

### Ethical considerations

The study complied with all ethical requirements of Brazilian National Health Council *(Conselho Nacional de Saúde,* CNS) resolution #466/2012 and was approved by the Ethics and Research Committee of Christus University Center (approval #43405315.3.0000.5049).

## RESULTS

Based on the analysis, six variables were statistically significant *(P* < 0.05) and were thus selected as indicators for the model: tourism, population density, percentage of urbanization, dropout rate between MMR dose 1 and 2, proportion of population covered by CHAs, and proportion of population covered by Family Health Strategy (FHS) teams (Table 1). Table 2 provides details about the scores and criteria for each indicator.

The model showed a sensitivity of 94.7% for concordance between municipalities classified as “high risk” and “very high risk” and those that had an epidemic between 2013 and 2015 in Ceará. Two of the 184 municipalities (5.3%) were classified as “low risk,” and had one confirmed case each, but the source of infection was not the municipality of residence, so both cases were considered imported. Among the 38 municipalities that had an epidemic, 76% (29/38) were classified as “high risk” and “very high risk”; the 146 municipalities that did not report cases 42.5% (62/146) had a “medium risk” classification *(P* < 0.0002).

**TABLE 2.** Description, cutoff points, and scores for health-related variables assessed as municipality risk factors for reintroduction/transmission of measles in a post-elimination scenario based on their association with the presence or absence of confirmed measles cases in 184 municipalities during an epidemic in Ceará, Brazil, 2013–2015

Variables^a^	Description	Cutoff point	Score
First extract of analysis			
Dropout rate between MMR^b^dose 1 and 2	Indicates the accumulation of the susceptible population between MMR dose 1 and 2	≥ 5%	Municipalities with a dropout rate ≥ 5%: 13 points Municipalities with a dropout rate < 5%: 0 points
Tourism	Indicates the possibility of an increase in foreign visitors. The classifications for different levels of tourism of Brazil's Ministry of Tourism (A-E) were used. These classifications (categories) are based on the number of establishments and workers involved in tourism in each municipality.	Categories A, B, and C: international tourism Categories D and E: national and local tourism	Municipalities with categories A, B, and C: 13 points Municipalities with categories D and E: 0 points Municipalities without any tourist areas: 0 points
Population density index	Indicates accumulation or dispersion of population within an area. To categorize each municipality, the state median (56 people per km2, in Ceará state) was used.	High density: ≥ 56 per km2 Low density: < 56 per km2	Municipalities with high density (≥ 56 per km2): 13 points Municipalities with low density < 56 per km2): 0 points
Urbanization	Indicates the growth of a city due to the increase of people. To categorize each municipality, the minimum proportion of residences in urban areas of the Brazilian Institute of Geography and Statistics (IBGE) (≥ 50) was used.	High urbanization: ≥ 50 Lower urbanization: < 50	Municipalities with high urbanization (≥ 50): 13 points Municipalities with low urbanization < 50): 0 points
Family Health Strategy (FHS) coverage	The presence of this strategy in the municipalities facilitates the implementation of a timely response (treatment and vaccination) in the community before any suspected cases of measles. The minimum proportion per municipality, according to the Ministry of Health (≥ 70%), was used.	High FHS coverage: ≥ 70% Low FHS coverage: < 70%	Municipalities with high FHS coverage (≥ 70%): 0 points Municipalities with low FHS coverage < 70%): 13 points
Community Health Agent (CHA) coverage	The presence of CHAs in the municipalities denoted permanent community surveillance to identify and report suspected measles cases in a timely manner. As a cutoff point, the minimum proportion of the Ministry of Health (≥ 80%) assigned to each municipality was used.	High CHA coverage: ≥ 80% Low CHA coverage: < 80%	Municipalities with high CHA coverage (≥ 80%): 0 points Municipalities with low CHA coverage < 80%): 14 pointsc^c^
Second extract of analysis			
MMR dose 1 coverage	Percentage of 1-year-old children vaccinated with MMR dose 1. The standard value (proportion) of the Ministry of Health and PAHO/WHO of ≥ 95% coverage was used.	High vaccination coverage for MMR dose 1: ≥ 95% Low vaccination coverage for MMR dose 1: < 95%	Municipalities with high coverage (≥ 95%): 0 points Municipalities with low coverage < 95%): 7 points
Reporting rate for exanthematic diseases	Indicates sensitivity of the municipality's surveillance system to capture and report suspected cases of measles or rubella. The standard value of the Ministry of Health and PAHO/WHO of at least 2 suspected cases reported per 100 000 inhabitants was used.	Notification rate ≥ 2 cases per 100 000 population	Municipalities with a rate ≥ 2 suspected cases per 100 000 inhabitants: 0 points Municipalities with a rate < 2 suspected cases per 100 000 inhabitants: 7 points
Presence of vulnerability conditions	The following vulnerability conditions were used: 1) border with other countries; 2) favelas (shanty towns); 3) violence; 4) indigenous communities; 5) population resistant to vaccination; 6) difficult geographic access; and 7) areas with trade fairs and mass events.	Vulnerability: presence of at least one condition	Municipalities with vulnerability: 7 points Municipalities without vulnerability: 0 points

***Source:*** Prepared by the authors based on the study results.

a Information about the variables used in the model are accessible online from the following sources: 1) the information system of Brazil’s publicly funded health care system, DATASUS(Departamento de Informática do Sistema Único de Saúde), the entity responsible for collecting, processing, and disseminating health information; 2) the National Immunization Information System (Sistema de Informação do Programa Nacional de Imunização, SI-PNI); 3) the Notification of Injury Information System (Sistema de Informação de Agravos de Notificação,SINAN); 4) the Brazilian Institute of Geography and Statistics (Instituto Brasileiro de Geografia e Estatística, IBGE); 5) Ceará Research Institute on Economic Strategy (Instituto de Pesquisa e Estratégia Econ ô mica do Ceará, IPECE); 6) the Latin American School of Social Sciences (Facultad Latinoamericana de Ciencias Sociales, FLACSO) (the “Violence Map”); 7) the Brazilian Ministry of Tourism (the “Tourism Map”); and 8) the Department of Primary Care of the Brazilian Ministry of Health (Departamento de Atenção Básica, Secretaria de Atenção à Saúde, DAB-MS).

b Measles-mumps-rubella vaccine.

c This variable had a higher odds ratio (7.22) so was worth 14 points.

## DISCUSSION

The model presented in this study used data on the pre-epidemic scenario in Ceará, Brazil, in an effort to reproduce the characteristics of areas with measles cases after elimination had been achieved. The risk analysis generated by the model was validated using data from the epidemic that occurred in Ceará between 2014 and 2015. The objective was to establish a relationship between certain characteristics of the municipalities and health systems and occurrence of the epidemic. The model presented here could be used as a tool for strategic planning to maintain, monitor, and sustain measles elimination efforts in the Americas.

**FIGURE 2.**
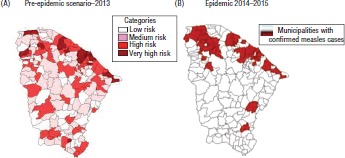
Predicted level of risk (“low,” “medium,” “high,” and “very high”) for measles reintroduction/transmission in 184 municipalities, based on the results of a proposed risk model, using data from the pre-epidemic scenario (A), and municipalities with confirmed measles cases one year later (B), Ceará, Brazil, 2013–2015

***Source:*** Prepared by the authors based on the study results.

All 184 municipalities included in the analysis scored poorly for the health system quality indicators related to immunization programs and epidemiological surveillance. If specific actions were implemented to improve the quality of these health system indicators, risk of measles reintroduction and transmission in these municipalities may decrease, even if deficits in all of the health-related indicators studied here can not be addressed through interventions due to resource constraints. Specific actions may also be taken to address some of the indi-cators unrelated to the health system, such as tourism and population density. For example, the authors suggest that 1) municipalities with a strong potential for tourism ensure that all professionals working in the tourism sector are vaccinated with two doses of the MMR; 2) municipalities with high rates of urbanization consider an alternative schedule for vaccinating the target population, given the dynamics of large urban centers; and 3) municipalities with high demographic densities consider microscenarios (community plans) for immunization to help prevent the accumulation of susceptible populations.

Various tools have been developed to predict the future occurrence of diseases in a population, including prediction models developed for breast cancer, in which screening or chemoprophylaxis may be considered useful for those at high risk, some of which are relevant not only for clinical decision-making but also for estimating overall health costs (14–20). Studies describing the application of risk stratification and prediction models suggest that there is strong evidence for the use of these predictive models with administrative and clinical data for patients with chronic disease (21–23).

In Ireland, using predictive models and risk stratification, certain actions were incorporated in primary care and hospital admissions were reduced (24). Other studies have shown efficacy in customizing and developing models or tools that meet the needs of a given health system—including predictive models for communicable diseases that consider the infectivity potential of the etiological agent and the level of population immunity—and are predominantly based on scientific evidence drawn from models of dynamic disease transmission, and designed to support global efforts to control and eliminate immune-preventable diseases (10, 25–30).

Endemic transmission of measles virus in other parts of the world remains a risk for regions that have eliminated the disease, and until there is a disruption of virus transmission worldwide, the possibility of importation of measles cases and occurrence of outbreaks remains. Although the elimination of measles in the Americas was certified in 2016, sporadic reintroductions may result in new transmission chains (13) that spread according to the level of immunity of the resident population (21). Therefore, the main challenges to maintaining the elimination of measles are ensuring 1) sensitive surveillance; 2) an effective response to the importation of the wild virus; 3) homogeneous vaccination coverage (≥ 95%) in the municipalities; and 4) the elaboration of an integrated action plan, with periodic risk analysis (15, 31).

The risk of transmission of the measles virus is associated with vaccine coverage and characteristics of the municipality such as response capacity and epidemiologic surveillance. The ideal scenario is that all measles cases be readily identified by the health service and secondary cases avoided by implementing strategies that contain transmission in the community. Therefore, the variables related to the public health response and structure of the health services of the municipalities, along with the operational indicators, allowed for analysis of the risk of transmission of the measles virus in Ceará.

### Limitations

The limitations of this study were mainly due to the inability to validate the study method in other countries in the Americas that have achieved measles elimination (including those that had epidemics, such as Ecuador and Venezuela) and in other states in Brazil (e.g., Bahia, Paraíba, and Pernambuco). This limitation was due to lack of access to databases from the abovementioned countries and other states in Brazil. The limitation was not related to the study method, and the variables studied here are available for other countries of the Americas. Future validation studies could address this gap. Another limitation may be the quality of the analyzed data, which were secondary and collected for other purposes.

### Conclusions

Given the imminent risk of reintroduction of measles circulation in the post-elimination period in the Americas, this model may be useful in identifying areas at greater risk for reintroduction and continued transmission of measles. Knowledge of vulnerable areas could trigger appropriate surveillance and monitoring to prevent sustained transmission.

### Disclaimer.

Authors hold sole responsibility for the views expressed in the manuscript, which may not necessarily reflect the opinion or policy of the RPSP/PAJPH or the Pan American Health Organization (PAHO).

## Footnotes

a The tool does not allow for measuring the risk of reintroduction of the virus in areas that have already eliminated the disease because the data measured in the study include the public health response to cases and the population profile affected by measles.
